# Occupational disorder of cobalt and indium in the crystal structure of Co_5_In_3_SbSi_2_O_16_

**DOI:** 10.1107/S2056989026010960

**Published:** 2026-10-23

**Authors:** Tobias Wolflehner, Matthias Weil

**Affiliations:** a Institute for Chemical Technologies and Analytics, Division of Applied Solid State Chemistry, Getreidemarkt 9/E164-05-1, 1060 Vienna, Austria; University of Aberdeen, United Kingdom

**Keywords:** crystal structure, co-occupation, isotypism, framework structure, structure comparison

## Abstract

Penta­cobalt(II) triindium(III) anti­mony(V) disilicon(VI) hexa­deca­oxide, Co_5_In_3_SbSi_2_O_16_, was obtained as a single-crystalline by-product through a reaction with the silica glass ampoule during a chemical vapor transport reaction. It crystallizes isotypically with Co_8_TeAs_2_O_16_ in space group *Cmce*, with two (out of four) Co sites co-occupied by In, the Te site replaced by Sb, and the As site replaced by Si. The crystal structure of Co_5_In_3_SbSi_2_O_16_ consists of [CoO_6_], [(In,Co)O_6_], and [SbO_6_] octa­hedra, and [CoO_4_] and [SiO_4_] tetra­hedra, fused by vertex- and edge-sharing into a complex three-dimensional framework.

## Abstract 2

Co_5_In_3_SbSi_2_O_16_ is isotypic with Co_8_TeAs_2_O_16_ and comprises [CoO_6_], [(In,Co)O_6_], [SbO_6_], [CoO_4_] and [SiO_4_] coordination polyhedra as principal building units.

## 1. Chemical context

First-row divalent transition-metal (*M*^II^) anti­monates with composition *M*_2_InSbO_6_ have a corundum-related crystal structure and exhibit inter­esting magnetic properties, including frustrated magnetism for Mn_2_InSbO_6_ (Ivanov *et al.*, 2011 ▸), long-range incommensurate anti­ferromagnetic order at low temperatures for Ni_2_InSbO_6_ (Ivanov *et al.*, 2013 ▸), or, depending on the modification of Co_2_InSbO_6_, a sharp ferrimagnetic transition at 65 K (Ji *et al.*, 2022 ▸). The relevant materials were produced as powder samples *via* solid-state reactions, either at normal pressure (*M* = Ni, Mn) or under high-pressure conditions (*M* = Co). Since chemical vapour transport reactions (Binnewies *et al.*, 2012 ▸) had been successfully applied for single crystal growth of Ni_2_InSbO_6_ in the millimetre size range (Weil *et al.*, 2014 ▸), an attempt was made to apply this method to crystal growth of Co_2_InSbO_6_. The crystals obtained during one of these experiments did not have the desired composition, but also contained silicon as an additional element, which resulted from a reaction with the silica glass ampoule. This article reports on the corresponding crystal structure of Co_5_In_3_SbSi_2_O_16_, (**I**).

## 2. Structural commentary

Compound (**I**) is isotypic with Co_8_TeAs_2_O_16_ (Staack & Müller-Buschbaum, 1997 ▸). It comprises two Co sites (Co1: multiplicity 8, Wyckoff letter *f*, site symmetry *m*..; Co2: 8 *e*, .2.), two mixed-occupied (In/Co) sites (In1/Co3: 8 *f*; In2/Co4: 8 *e*), one Si (Si1: 8 *f*), one Sb (Sb1: 4 *a*, 2/*m*..) and six O sites (O2–O5: 8 *f*; O1, O6: 16 *g*, 1) in the asymmetric unit. Compared with Co_8_TeAs_2_O_16_, sites Co3 and Co4 of Co_5_In_3_SbSi_2_O_16_ are co-occupied by In, the Te site is replaced by Sb, and the As site by Si. Co-occupation of sites by Co and In was also observed for different modifications of Co_2_InSbO_6_ (Ji *et al.*, 2022 ▸) or Co_2_InBO_5_ and Co_3–x_In_x_BO_5_ solid solutions (Cai *et al.*, 2014 ▸).

The principal building units in the crystal structure of (**I**) are [Co1O_4_] and [Si1O_4_] tetra­hedra and [Co2O_6_], [(In1,Co3)O_6_], [(In2,Co4)O_6_] and [Sb1O_6_] octa­hedra. Ranges and averages of bond lengths in these polyhedra comply with literature values and are summarized in Table 1 ▸.

The rather complex crystal structure of (**I**) is made up from vertex- and edge-sharing of the building units and is displayed in Fig. 1 ▸. As the structural details have already been discussed at length in connection with isotypic Co_8_TeAs_2_O_16_ (Staack & Müller-Buschbaum, 1997 ▸), we will limit ourselves to the most notable features: The [Sb1O_6_] octa­hedra are isolated from others, and a {Co1Si1O_7_} double tetra­hedron is formed *via* a bridging O atom (O2) and is also isolated from other double tetra­hedra. These entities are linked by [100] chains of edge-sharing [CoO_6_] and [(In,Co)O_6_] octa­hedra into a framework structure; a fragment of this is shown in Fig. 2 ▸.

Bond-valence-sum (BVS) calculations (Brown, 2002 ▸) were carried out with the *ECoN21* program (Ilinca, 2022 ▸) to validate the structure model. The calculated bond-valence sums (Table 2 ▸) of Co1, Co2, Sb1, Si1 and the six O atoms are close to the expected values (in parentheses) for Co (2), Sb (5), Si (4) and O (2) atoms. The mixed-occupied (In1,Co3) and (In2,Co4) sites exhibit BVS values between 2 and 3, which is consistent with the formal charges of +2 for Co and +3 for In, as well as the different In:Co ratio. The strongest deviation from expectation pertains to O4, which is bonded to five metal cations [2Co, 3(In,Co)] and not to the higher-charged Si or Sb atoms. The global instability index (GII) of 0.11 valence units indicates a stable and not particularly strained crystal structure (Salinas-Sanchez *et al.*, 1992 ▸; Brown, 2009 ▸).

Numerical data from a qu­anti­tative comparison of the isotypic structures of (**I**) and Co_8_TeAs_2_O_16_, carried out using the *compstru* program (de la Flor *et al.*, 2016 ▸), which is available on the Bilbao Crystallographic Server (Aroyo *et al.*, 2006 ▸), are summarized in Table 2 ▸. The low values for the degree of lattice distortion (0.0050) and the measure of similarity (0.027) indicate a high degree of similarity between the two crystal structures. The highest differences in atomic positions pertain to atoms O2 and O3, both of which are part of the silicate tetra­hedron and two [CoO_6_] (for the arsenate) or one [CoO_6_] and one [(In,Co)O_6_] octa­hedron (for the silicate). Inter­estingly, the differences for O4, which is bound to five octa­hedrally coordinated metal atoms [Co or (In,Co)] but not to the double tetra­hedron or the Sb (Te) atom (Table 2 ▸), are small.

## 3. Synthesis and crystallization

Co_5_In_3_SbSi_2_O_16_ formed serendipitously during phase-formation studies in the system Co/In/Sb/O aimed at a phase with composition Co_2_InSbO_6_ (Ji *et al.*, 2022 ▸) by using chemical vapour transport reactions (CVTR; Binnewies *et al.*, 2012 ▸). Stoichiometric amounts of Co_3_O_4_ (Alfa-Aesar, >99%), In_2_O_3_ (Merck KGaA, >99.5%) and Sb_2_O_5_ [Thermo Fisher Scientific, 99.998% (metals basis)] were weighted in an 1.333:1:1 ratio (altogether *ca*. 300 mg), intimidately ground in an agate mortar and combined with HgCl_2_ (60 mg) as the transport agent. The mixture was loaded in a silica ampoule (volume ca. 20 cm^3^) that was evacuated, sealed and placed in a two-zone furnace using a gradient 1223 → 1123 K for 4 days. Co_5_In_3_SbSi_2_O_16_ crystallized in the cooler sink side by reacting with the glass ampoule in form of sub-mm blue plates; brown crystals of preliminary composition Co_8_In_11_Sb_7_O_42_ were also present at the sink side. Powder X-ray diffraction of the bulk revealed an approximate phase ratio Co_5_In_3_SbSi_2_O_16_:Co_8_In_11_Sb_7_O_42_ of 1:9. At the source side, CoSb_2_O_6_ (Reimers *et al.*, 1989 ▸) and monoclinic InSbO_4_ (Wolflehner, 2026 ▸) were identified.

An attempt to synthesize Co_5_In_3_SbSi_2_O_16_ specifically under the same conditions as described above, using Co_3_O_4_, Sb_2_O_5_, In_2_O_3_ and SiO_2_ (Merck, purum) in the stoichiometric ratio 1.67:0.5:1.5:2, resulted in the formation of Co_5_In_3_SbSi_2_O_16_ crystals as the only product present in the sink region. In the source region, Co_5_In_3_SbSi_2_O_16_, CoSb_2_O_6_ and spinel-type Co_2.33_Sb_0.67_O_4_ (Dulac & Durif, 1960 ▸) were identified in an approximate ratio of 5:1:1 according to powder X-ray diffraction of the bulk.

## 4. Refinement

Crystal data, data collection and structure refinement details are summarized in Table 3 ▸. Starting atomic coordinates and the labelling scheme were taken from isotypic Co_8_As_2_TeO_16_ (Staack & Müller-Buschbaum, 1997 ▸). Initial refinement considering two Co, two In, one Sb, one Si and six O sites resulted in the composition Co_4_In_4_SbSi_2_O_16_, i.e. a charged compound under consideration of oxidation states of +II for Co, +III for In, +V for Sb and +IV for Si. Successive free refinement of the site occupation factors of the metal sites revealed full occupation for Co1, Co2, Sb1, Si1, but a minor under-occupation for In1 and a pronounced under-occupation for In2. To achieve charge neutrality, both sites were co-occupied with Co atoms in a fixed In1:Co3 ratio of 0.9:0.1 and In2:Co4 ratio of 0.6:0.4. The remaining maximum and minimum electron densities in the final model are located 0.96 and 0.76 Å, respectively, from Sb1.

## Supplementary Material

Crystal structure: contains datablock(s) I. DOI: 10.1107/S2056989026010960/hb8265sup1.cif

Structure factors: contains datablock(s) I. DOI: 10.1107/S2056989026010960/hb8265Isup2.hkl

CCDC reference: 2597010

Additional supporting information:  crystallographic information; 3D view; checkCIF report

## Figures and Tables

**Figure 1:**
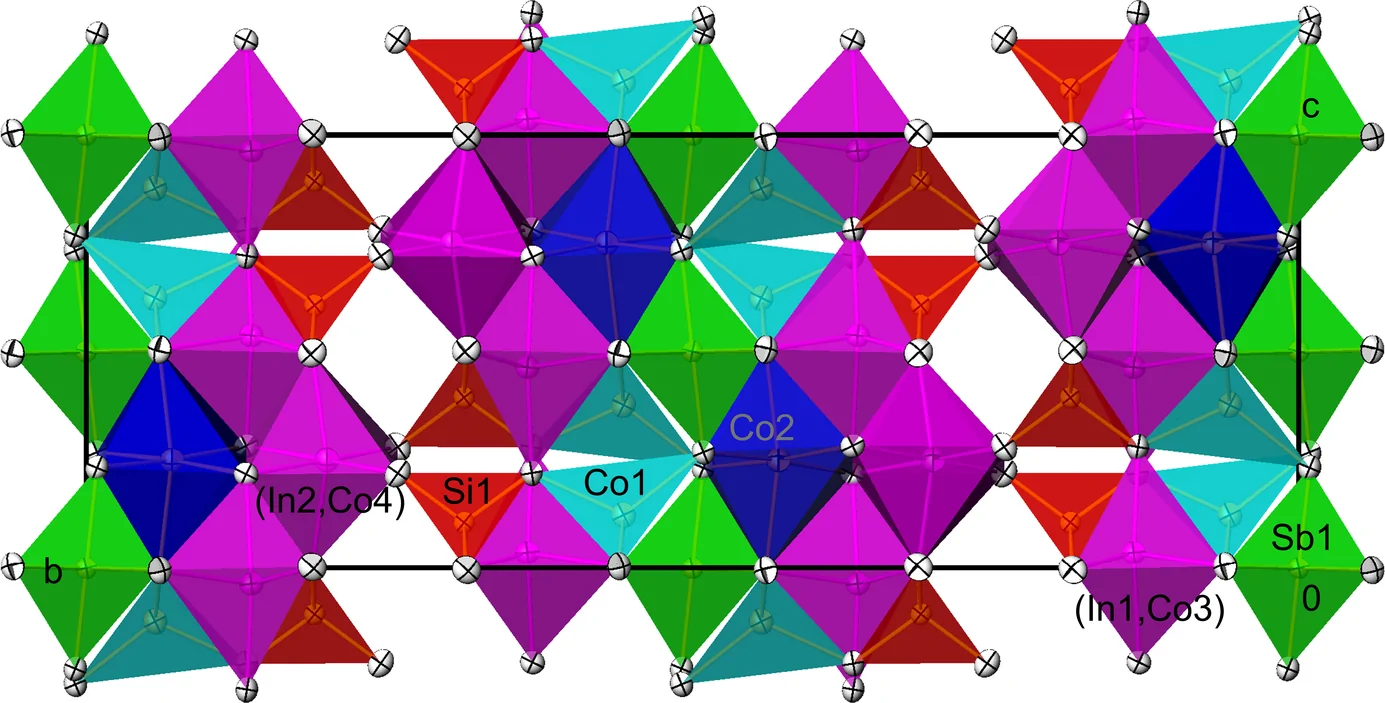
Polyhedral representation of the crystal structure of (**I**) in a view along [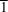
00]. Displacement ellipsoids are represented at the 90% probability level. Colour codes for polyhedra: Co in tetra­hedral coordination is turquoise, Co2 in octa­hedral coordination is blue, mixed (In,Co) sites in octa­hedral coordination are pink, anti­monate octa­hedra are green and silicate tetra­hedra are red.

**Figure 2:**
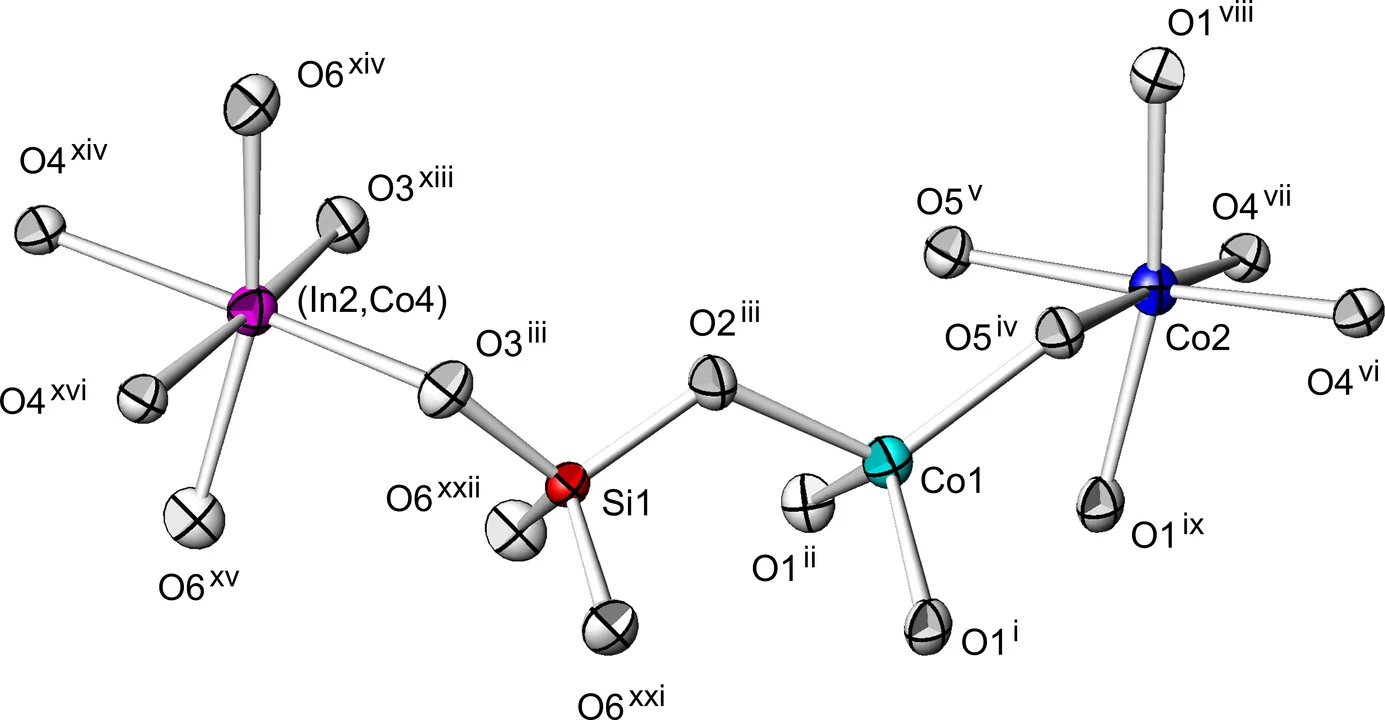
Fragment of the crystal structure showing the {Co1Si1O_7_} double tetra­hedron linked to [(In2,Co4)O_6_] and [Co2O_6_] octa­hedra. Displacement ellipsoids are as in Fig. 1 ▸. [Symmetry codes: (i) *x* − 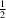, *y* + 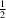, *z*; (ii) −*x* + 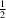, *y* + 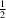, *z*; (iii) *x*, *y* + 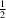, −*z* + 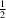; (iv) *x*, *y* − 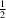, −*z* + 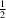; (v) *x* + 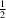, *y* − 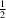, *z*; (vi) −*x*, −*y* + 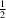, *z* − 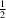; (vii) −*x* + 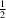, −*y* + 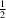, −*z* + 1; (viii) *x*, −*y* + 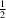, *z* + 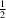; (ix) −*x* + 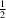, −*y* + 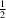, −*z*; (xiii) *x* + 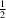, *y* + 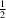, *z*; (xiv) −*x* + 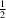, −*y* + 1, *z* − 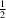; (xv) *x*, −*y* + 1, −*z* + 1; (xvi) −*x*, −*y* + 1, −*z* + 1; (xxi) *x* − 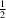, *y* + 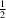, *z* − 1; (xxii) −*x* + 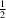, *y* + 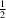, *z* − 1.]

**Table 1:** Details of the coordination by O atoms around the metal cations and results of BVS calculations

Site	Coordination number	Range of bond lengths (Å)	Mean bond length (Å)	Mean bond length (literature value) (Å)	BVS (valence units)
Co1	4	1.977 (2)–1.997 (3)	1.984	1.967 (22)^*c*^	1.86
Co2	6	2.128 (2)–2.1913 (19)	2.147	2.108 (62)^*c*^	1.82
(In1,Co3)^*a*^	6	2.078 (3)–2.2745 (19)	2.156	Co 2.108 (62)^*c*^, In 2.142 (42)^*d*^	2.73
(In2,Co4)^*b*^	6	2.084 (2)–2.219 (2)	2.137	Co 2.108 (62)^*c*^, In 2.142 (42)^*d*^	2.53
Sb1	6	1.9818 (19)–1.989 (3)	1.984	1.977 (34)^*d*^	4.94
Si1	4	1.622 (3)–1.648 (3)	1.630	1.625 (24)^*d*^	3.94
O1	4 [2Co+Sb+(In,Co)]				1.93
O2	3 [Si+Co+(In,Co)]				1.96
O3	3 [Si+2(In,Co)]				1.97
O4	5 [2Co+3(In,Co)]				1.80
O5	4 (Sb+3Co)				1.90
O6	3 [Si+2(In,Co)]				1.93

Notes: (*a*) In:Co ratio 0.9:0.1; (*b*) In:Co ratio 0.6:0.4; (*c*) Gagné & Hawthorne (2020 ▸); (*d*) Gagné & Hawthorne (2018 ▸).

**Table 2:** Absolute distances |u| (Å) of matching atom pairs, arithmetic mean (*d*_av_) of distances (Å), degree of lattice distortion (S) and measure of similarity (*δ*) in the isotypic crystal structures of Co_5_In_3_SbSi_2_O_16_ and Co_8_TeAs_2_O_16_

Co1	0.0523		O1	0.0526
Co2	0.0817		O2	0.1220
(In1,Co3)^*a*^/Co3	0.0943		O3	0.1195
(In2,Co4)^*b*^/Co4	0.1057		O4	0.0413
Sb1/Te1	0		O5	0.0243
Si1/As1	0.0489		O6	0.0709
				
S	0.0050			
*d*_av_	0.0694			
*δ*	0.027			

Notes: (*a*) In:Co ratio 0.9:0.1; (*b*) In:Co ratio 0.6:0.4.

**Table 3:** Experimental details

Crystal data	
Chemical formula	Co_5_In_3_SbSi_2_O_16_
*M*_r_	1073.04
Crystal system, space group	Orthorhombic, *Cmce*
Temperature (K)	293
*a*, *b*, *c* (Å)	6.1012 (4), 23.8586 (6), 8.5282 (18)
*V* (Å^3^)	1241.4 (3)
*Z*	4
Radiation type	Mo *K*α
μ (mm^−1^)	14.40
Crystal size (mm)	0.10 × 0.08 × 0.05

Data collection	
Diffractometer	Stoe STADIVARI
Absorption correction	Multi-scan (*LANA*; Koziskova *et al.*, 2016 ▸)
*T*_min_, *T*_max_	0.474, 0.874
No. of measured, independent and observed [*I* > 2σ(*I*)] reflections	6885, 1580, 1320
*R*_int_	0.032
(sin θ/λ)_max_ (Å^−1^)	0.834

Refinement	
*R*[*F*^2^ > 2σ(*F*^2^)], *wR*(*F*^2^), *S*	0.024, 0.056, 1.01
No. of reflections	1580
No. of parameters	75
Δρ_max_, Δρ_min_ (e Å^−3^)	1.54, −2.23

Computer programs: *X-AREA* (Folkers-Karlsson *et al.*, 2026 ▸), *SHELXT* (Sheldrick, 2015*a* ▸), *SHELXL* (Sheldrick, 2015*b* ▸), *ATOMS* (Dowty, 2006 ▸) and *publCIF* (Westrip, 2010 ▸).

## supplementary crystallographic information

### Pentacobalt(II) triindium(III) antimony(V) disilicon(VI) hexadecaoxide Crystal data

**Table:**

Co_5_In_3_SbSi_2_O_16_	*D*_x_ = 5.741 Mg m^−^^3^
*M**_r_* = 1073.04	Mo *K*α radiation, λ = 0.71073 Å
Orthorhombic, *C**m**c**e*	Cell parameters from 14765 reflections
*a* = 6.1012 (4) Å	θ = 3.4–36.5°
*b* = 23.8586 (6) Å	µ = 14.40 mm^−^^1^
*c* = 8.5282 (18) Å	*T* = 293 K
*V* = 1241.4 (3) Å^3^	Plate, red
*Z* = 4	0.10 × 0.08 × 0.05 mm
*F*(000) = 1956	

### Pentacobalt(II) triindium(III) antimony(V) disilicon(VI) hexadecaoxide Data collection

**Table:**

Stoe STADIVARI diffractometer	1320 reflections with *I* > 2σ(*I*)
Detector resolution: 13.33 pixels mm^-1^	*R*_int_ = 0.032
rotation method, ω scans	θ_max_ = 36.4°, θ_min_ = 3.4°
Absorption correction: multi-scan (*LANA*; Koziskova *et al.*, 2016)	*h* = −3→10
*T*_min_ = 0.474, *T*_max_ = 0.874	*k* = −39→35
6885 measured reflections	*l* = −6→14
1580 independent reflections	

### Pentacobalt(II) triindium(III) antimony(V) disilicon(VI) hexadecaoxide Refinement

**Table:**

Refinement on *F*^2^	75 parameters
Least-squares matrix: full	0 restraints
*R*[*F*^2^ > 2σ(*F*^2^)] = 0.024	*w* = 1/[σ^2^(*F*_o_^2^) + (0.033*P*)^2^] where *P* = (*F*_o_^2^ + 2*F*_c_^2^)/3
*wR*(*F*^2^) = 0.056	(Δ/σ)_max_ = 0.001
*S* = 1.01	Δρ_max_ = 1.54 e Å^−^^3^
1580 reflections	Δρ_min_ = −2.23 e Å^−^^3^

### Pentacobalt(II) triindium(III) antimony(V) disilicon(VI) hexadecaoxide Special details

**Table:**

Geometry. All esds (except the esd in the dihedral angle between two l.s. planes) are estimated using the full covariance matrix. The cell esds are taken into account individually in the estimation of esds in distances, angles and torsion angles; correlations between esds in cell parameters are only used when they are defined by crystal symmetry. An approximate (isotropic) treatment of cell esds is used for estimating esds involving l.s. planes.

### Pentacobalt(II) triindium(III) antimony(V) disilicon(VI) hexadecaoxide Fractional atomic coordinates and isotropic or equivalent isotropic displacement parameters (Å^2^)

**Table:**

	*x*	*y*	*z*	*U*_iso_*/*U*_eq_	Occ. (<1)
Co1	0.000000	0.55684 (2)	0.12353 (6)	0.00899 (10)	
Co2	0.250000	0.42916 (2)	0.250000	0.00861 (10)	
Co3	0.000000	0.13625 (2)	0.03647 (3)	0.00856 (6)	0.1
In1	0.000000	0.13625 (2)	0.03647 (3)	0.00856 (6)	0.9
Co4	0.250000	0.80155 (2)	0.250000	0.00934 (7)	0.4
In2	0.250000	0.80155 (2)	0.250000	0.00934 (7)	0.6
Si1	0.000000	0.68839 (4)	0.10629 (12)	0.00706 (17)	
Sb1	0.000000	0.000000	0.000000	0.00677 (7)	
O1	0.2222 (3)	0.06057 (8)	0.0046 (2)	0.0096 (3)	
O2	0.000000	0.13256 (11)	0.2799 (3)	0.0108 (5)	
O3	0.000000	0.24301 (12)	0.2804 (3)	0.0106 (5)	
O4	0.000000	0.13194 (11)	0.7828 (3)	0.0089 (5)	
O5	0.000000	0.99103 (12)	0.2319 (3)	0.0098 (5)	
O6	0.2821 (3)	0.18631 (8)	0.9968 (2)	0.0107 (4)	

### Pentacobalt(II) triindium(III) antimony(V) disilicon(VI) hexadecaoxide Atomic displacement parameters (Å^2^)

**Table:**

	*U*^11^	*U*^22^	*U*^33^	*U*^12^	*U*^13^	*U*^23^
Co1	0.00738 (19)	0.0093 (2)	0.0103 (2)	0.000	0.000	0.00064 (18)
Co2	0.00728 (19)	0.0091 (2)	0.0095 (2)	0.000	0.00118 (16)	0.000
Co3	0.00825 (10)	0.00913 (12)	0.00829 (12)	0.000	0.000	−0.00009 (9)
In1	0.00825 (10)	0.00913 (12)	0.00829 (12)	0.000	0.000	−0.00009 (9)
Co4	0.00864 (13)	0.00895 (15)	0.01044 (15)	0.000	−0.00114 (10)	0.000
In2	0.00864 (13)	0.00895 (15)	0.01044 (15)	0.000	−0.00114 (10)	0.000
Si1	0.0067 (4)	0.0072 (4)	0.0073 (4)	0.000	0.000	−0.0004 (4)
Sb1	0.00580 (12)	0.00698 (14)	0.00754 (15)	0.000	0.000	0.00012 (11)
O1	0.0072 (7)	0.0092 (9)	0.0123 (9)	−0.0016 (6)	−0.0006 (6)	0.0005 (8)
O2	0.0142 (12)	0.0076 (12)	0.0106 (13)	0.000	0.000	−0.0005 (10)
O3	0.0086 (10)	0.0106 (12)	0.0124 (13)	0.000	0.000	0.0023 (10)
O4	0.0085 (10)	0.0094 (12)	0.0089 (12)	0.000	0.000	0.0004 (10)
O5	0.0113 (11)	0.0083 (12)	0.0098 (12)	0.000	0.000	−0.0007 (10)
O6	0.0064 (7)	0.0130 (10)	0.0128 (9)	−0.0016 (6)	−0.0016 (6)	0.0010 (8)

### Pentacobalt(II) triindium(III) antimony(V) disilicon(VI) hexadecaoxide Geometric parameters (Å, º)

**Table:**

Co1—O1^i^	1.977 (2)	In2—O3^iii^	2.084 (2)
Co1—O1^ii^	1.977 (2)	In2—O3^xiii^	2.084 (2)
Co1—O2^iii^	1.986 (3)	In2—O6^xiv^	2.1340 (19)
Co1—O5^iv^	1.997 (3)	In2—O6^xv^	2.1340 (19)
Co2—O5^v^	2.128 (2)	In2—O4^xiv^	2.219 (2)
Co2—O5^iv^	2.128 (2)	In2—O4^xvi^	2.219 (2)
Co2—O4^vi^	2.128 (2)	Sb1—O1^xvii^	1.9818 (19)
Co2—O4^vii^	2.128 (2)	Sb1—O1^xii^	1.9818 (19)
Co2—O1^viii^	2.1913 (19)	Sb1—O1^xviii^	1.9818 (19)
Co2—O1^ix^	2.1913 (19)	Sb1—O1	1.9818 (19)
In1—O2	2.078 (3)	Sb1—O5^xix^	1.989 (3)
In1—O6^x^	2.1220 (18)	Sb1—O5^xx^	1.989 (3)
In1—O6^xi^	2.1220 (18)	Si1—O3^iii^	1.622 (3)
In1—O4^x^	2.165 (3)	Si1—O6^xxi^	1.6252 (19)
In1—O1	2.2744 (19)	Si1—O6^xxii^	1.6252 (19)
In1—O1^xii^	2.2745 (19)	Si1—O2^iii^	1.648 (3)
			
O1^i^—Co1—O1^ii^	118.00 (11)	O3^iii^—Si1—O6^xxii^	111.51 (10)
O1^i^—Co1—O2^iii^	99.91 (8)	O6^xxi^—Si1—O6^xxii^	109.78 (14)
O1^ii^—Co1—O2^iii^	99.91 (8)	O3^iii^—Si1—O2^iii^	107.36 (15)
O1^i^—Co1—O5^iv^	110.62 (7)	O6^xxi^—Si1—O2^iii^	108.28 (10)
O1^ii^—Co1—O5^iv^	110.62 (7)	O6^xxii^—Si1—O2^iii^	108.28 (10)
O2^iii^—Co1—O5^iv^	117.34 (12)	O1^xvii^—Sb1—O1^xii^	93.67 (11)
O5^v^—Co2—O5^iv^	92.17 (11)	O1^xvii^—Sb1—O1^xviii^	86.33 (11)
O5^v^—Co2—O4^vi^	176.59 (10)	O1^xii^—Sb1—O1^xviii^	180.00 (11)
O5^iv^—Co2—O4^vi^	87.25 (7)	O1^xvii^—Sb1—O1	180.0
O5^v^—Co2—O4^vii^	87.25 (7)	O1^xii^—Sb1—O1	86.33 (11)
O5^iv^—Co2—O4^vii^	176.59 (10)	O1^xviii^—Sb1—O1	93.67 (11)
O4^vi^—Co2—O4^vii^	93.54 (11)	O1^xvii^—Sb1—O5^xix^	93.38 (8)
O5^v^—Co2—O1^viii^	92.86 (9)	O1^xii^—Sb1—O5^xix^	86.62 (8)
O5^iv^—Co2—O1^viii^	78.17 (9)	O1^xviii^—Sb1—O5^xix^	93.38 (8)
O4^vi^—Co2—O1^viii^	83.73 (9)	O1—Sb1—O5^xix^	86.62 (8)
O4^vii^—Co2—O1^viii^	105.22 (10)	O1^xvii^—Sb1—O5^xx^	86.62 (8)
O5^v^—Co2—O1^ix^	78.17 (9)	O1^xii^—Sb1—O5^xx^	93.38 (8)
O5^iv^—Co2—O1^ix^	92.86 (9)	O1^xviii^—Sb1—O5^xx^	86.62 (8)
O4^vi^—Co2—O1^ix^	105.22 (10)	O1—Sb1—O5^xx^	93.38 (8)
O4^vii^—Co2—O1^ix^	83.73 (9)	O5^xix^—Sb1—O5^xx^	180.00 (17)
O1^viii^—Co2—O1^ix^	167.15 (10)	Co1^v^—O1—Sb1	124.31 (10)
O2—In1—O6^x^	100.54 (7)	Co1^v^—O1—Co2^ix^	116.55 (9)
O2—In1—O6^xi^	100.54 (7)	Sb1—O1—Co2^ix^	96.61 (8)
O6^x^—In1—O6^xi^	108.41 (10)	Co1^v^—O1—In1	119.02 (9)
O2—In1—O4^x^	174.85 (10)	Sb1—O1—In1	99.99 (8)
O6^x^—In1—O4^x^	82.40 (7)	Co2^ix^—O1—In1	94.35 (7)
O6^xi^—In1—O4^x^	82.40 (7)	Si1^iv^—O2—Co1^iv^	119.41 (17)
O2—In1—O1	94.93 (8)	Si1^iv^—O2—In1	123.65 (16)
O6^x^—In1—O1	86.81 (7)	Co1^iv^—O2—In1	116.94 (14)
O6^xi^—In1—O1	155.68 (8)	Si1^iv^—O3—Co4^iv^	127.76 (8)
O4^x^—In1—O1	80.96 (7)	Si1^iv^—O3—Co4^xxiii^	127.76 (8)
O2—In1—O1^xii^	94.93 (8)	Co4^iv^—O3—Co4^xxiii^	94.07 (12)
O6^x^—In1—O1^xii^	155.68 (8)	Co2^xxiv^—O4—Co2^vii^	91.56 (11)
O6^xi^—In1—O1^xii^	86.81 (7)	Co2^xxiv^—O4—Co3^xxv^	99.44 (9)
O4^x^—In1—O1^xii^	80.96 (7)	Co2^vii^—O4—Co3^xxv^	99.44 (9)
O1—In1—O1^xii^	73.18 (10)	Co2^xxiv^—O4—Co4^xxvi^	164.99 (15)
O3^iii^—In2—O3^xiii^	95.85 (12)	Co2^vii^—O4—Co4^xxvi^	88.89 (2)
O3^iii^—In2—O6^xiv^	98.43 (9)	Co3^xxv^—O4—Co4^xxvi^	95.28 (10)
O3^xiii^—In2—O6^xiv^	92.02 (9)	Co2^xxiv^—O4—Co4^xvi^	88.89 (2)
O3^iii^—In2—O6^xv^	92.02 (9)	Co2^vii^—O4—Co4^xvi^	164.99 (15)
O3^xiii^—In2—O6^xv^	98.43 (9)	Co3^xxv^—O4—Co4^xvi^	95.28 (10)
O6^xiv^—In2—O6^xv^	164.39 (11)	Co4^xxvi^—O4—Co4^xvi^	86.85 (10)
O3^iii^—In2—O4^xiv^	176.38 (8)	Sb1^xxvii^—O5—Co1^iii^	121.97 (15)
O3^xiii^—In2—O4^xiv^	87.73 (7)	Sb1^xxvii^—O5—Co2^i^	98.44 (10)
O6^xiv^—In2—O4^xiv^	80.88 (9)	Co1^iii^—O5—Co2^i^	120.04 (9)
O6^xv^—In2—O4^xiv^	87.96 (9)	Sb1^xxvii^—O5—Co2^iii^	98.44 (10)
O3^iii^—In2—O4^xvi^	87.73 (7)	Co1^iii^—O5—Co2^iii^	120.04 (9)
O3^xiii^—In2—O4^xvi^	176.38 (8)	Co2^i^—O5—Co2^iii^	91.56 (11)
O6^xiv^—In2—O4^xvi^	87.96 (9)	Si1^xxviii^—O6—Co3^xxv^	126.11 (11)
O6^xv^—In2—O4^xvi^	80.88 (9)	Si1^xxviii^—O6—Co4^xxvi^	129.60 (11)
O4^xiv^—In2—O4^xvi^	88.68 (10)	Co3^xxv^—O6—Co4^xxvi^	99.15 (7)
O3^iii^—Si1—O6^xxi^	111.51 (10)		

Symmetry codes: (i) *x*−1/2, *y*+1/2, *z*; (ii) −*x*+1/2, *y*+1/2, *z*; (iii) *x*, *y*+1/2, −*z*+1/2; (iv) *x*, *y*−1/2, −*z*+1/2; (v) *x*+1/2, *y*−1/2, *z*; (vi) −*x*, −*y*+1/2, *z*−1/2; (vii) −*x*+1/2, −*y*+1/2, −*z*+1; (viii) *x*, −*y*+1/2, *z*+1/2; (ix) −*x*+1/2, −*y*+1/2, −*z*; (x) *x*, *y*, *z*−1; (xi) −*x*, *y*, *z*−1; (xii) −*x*, *y*, *z*; (xiii) *x*+1/2, *y*+1/2, *z*; (xiv) −*x*+1/2, −*y*+1, *z*−1/2; (xv) *x*, −*y*+1, −*z*+1; (xvi) −*x*, −*y*+1, −*z*+1; (xvii) −*x*, −*y*, −*z*; (xviii) *x*, −*y*, −*z*; (xix) −*x*, −*y*+1, −*z*; (xx) *x*, *y*−1, *z*; (xxi) *x*−1/2, *y*+1/2, *z*−1; (xxii) −*x*+1/2, *y*+1/2, *z*−1; (xxiii) *x*−1/2, *y*−1/2, *z*; (xxiv) −*x*, −*y*+1/2, *z*+1/2; (xxv) *x*, *y*, *z*+1; (xxvi) −*x*+1/2, −*y*+1, *z*+1/2; (xxvii) *x*, *y*+1, *z*; (xxviii) *x*+1/2, *y*−1/2, *z*+1.
